# Reflections around ‘the cautionary use’ of the h-index: response to Teixeira da Silva and Dobránszki

**DOI:** 10.1007/s11192-018-2683-0

**Published:** 2018-03-09

**Authors:** Rodrigo Costas, Thomas Franssen

**Affiliations:** Centre for Science and Technology Studies (CWTS), Leiden University, Leiden, The Netherlands

**Keywords:** h-index, Research evaluation, Individual level bibliometric indicators

## Abstract

In a recent Letter to the Editor Teixeira da Silva and Dobránszki (2018) present a discussion of the issues regarding the h-index as an indicator for the evaluation of individual scholars, particularly in the current landscape of the proliferation of online sources that provide individual level bibliometric indicators. From our point of view, the issues surrounding the h-index go far beyond the problems mentioned by TSD. In this letter we provide some overview of this, mostly by expanding TSD’s original argument and discussing more conceptual and global issues related to the indicator, particularly in the outlook of a strong proliferation of online sources providing individual researcher indicators. Our discussion focuses on the h-index and the profusion of sources providing it, but we emphasize that many of our points are of a more general nature, and would be equally relevant for other indicators that reach the same level of popularity as the h-index.

## Introduction

In a recent Letter to the Editor Teixeira da Silva and Dobránszki (2018) (hereafter TSD) present a discussion of the issues regarding the h-index as an indicator for the evaluation of individual scholars, particularly in the current landscape of the proliferation of online sources that provide individual-level bibliometric indicators.

TSD’s argument starts with a brief description of the h-index (Hirsch 2005) illustrating their own experience in being confronted with different versions of the indicator coming from different bibliographic databases and online platforms. This motivated the authors to discuss some of the issues surrounding the h-index, including among others, its size dependency, its lack of field-normalization and its dependency on diverse databases for its calculation (raising issues around their coverage, data quality, etc.). TSD’s letter can be welcomed as yet another warning about the limitations and dangers of the h-index.

However, what probably is more disputable about TSD’s letter is not what it says, but what it does not say. In fact, the letter leaves the impression that if some technical issues are solved in these online platforms, their h-indexes will be useful for research evaluation. From our point of view, the issues surrounding the h-index go far beyond the problems mentioned by TSD. In this letter we provide some overview of this, mostly by expanding TSD’s original argument and discussing more conceptual and global issues related to the indicator, particularly in the outlook of a strong proliferation of online sources providing individual researcher indicators. Our discussion focuses on the h-index and the profusion of sources providing it, but we emphasize that many of our points are of a more general nature, and would be equally relevant for other indicators that reach the same level of popularity as the h-index.

The rest of this letter is structured as follows. In the next section we depict some of the most fundamental issues surrounding the h-index. In the second section, the current proliferation of sources providing h-indexes is addressed; and building on these two sections, the third section reflects on important warnings regarding the profusion of these h-indexes and their use for research evaluation. The letter ends with some final considerations on the use of individual-level bibliometrics in general.

## Issues of the h-index

The h-index has been strongly discussed and criticized nearly since the moment of its publication in 2005. Discussions around its size-dependency, inconsistency, biases, etc. have been frequent in the literature (Costas and Bordons 2007), together with suggestions of improvements or modifications (Egghe 2006; Egghe and Rousseau 2008). However, all these warnings and discussions did not prevent the h-index becoming a mainstream indicator and, as TSD illustrate, the indicator is often requested in research evaluations and is calculated and distributed by several online platforms. Probably its simplicity, easiness of calculation and broad availability across multiple platforms have been important factors contributing to the popularization of the h-index as an indicator to evaluate scholars’ performance.

From an analytical point of view, perhaps the only additional information provided by the h-index when compared to other common size-dependent bibliometric indicators (particularly the total number of publications [P] and the total number of citations [C]), is that it provides some rough indication about the spread of citations within the publication profile of an individual. Let’s suppose two researchers (A and B), both with 10 publications and 100 citations each, but A having one paper with 100 citations and the rest uncited (h-index = 1), and B receiving 10 citations in each of her papers (h-index = 10). The h-index would inform us that B has a more spread (even) distribution of citations as compared to A. Thus, anyone using the h-index must be aware of this predilection of the indicator for more distributed profiles of citations versus more concentrated ones. Moreover, even if A had published 5 papers of 20 citations each (h-index = 5), it would still have a lower h-index than B, illustrating how the h-index punishes *selectivity* (Costas and Bordons 2007, 2008). This shows how the h-index has a preference towards scholars who produce many moderately cited publications over those who prefer to produce a few high impact papers.

These examples illustrate how the h-index, like essentially any other indicator, incorporates specific choices and preferences. This directly challenges the idea of the h-index as a general (objective) indicator of individual scientific performance, which seems to be a quite common widespread idea in research evaluation practices.

## A profusion of platforms providing individual-level indicators

TSD’s letter raises and important issue: there is a proliferation of sources providing h-indexes and collecting bibliographic and citation data at the individual level. Typically, these new sources offer the promise of faster and easier performance evaluations of individual scholars. TSD mention Google Scholar, ResearchGate, Academia.edu and Loop, but the same goes for Microsoft Academic, AMiner, Scholar Universe or SemanticScholar.org. Many of these platforms usually offer the more traditional bibliometric indicators (P, C, h-index), as well as indicators of downloads/views, social media metrics and even more complex indicators such as the RG-score, citation velocity, highly influential citations, diversity or rising star, etc.

As pointed out by TSD, the proliferation of these sources confronts users with different (if not contradicting) results when analyzing the performance of scholars. Thus, users may be forced to choose one of the sources, for which the understanding of the limitations of each source is important. TSD point to the following issues in these sources: data curation, wrong data, inaccurate indicators, coverage, and the consideration of self-citations and retractions. We believe however, that there are also some other more fundamental issues:

### Lack of transparency and ‘black box’ nature

Most of these new sources do not disclose their size or coverage, and their limitations are unknown (Wouters and Costas 2012). None of them disclose information about the individuals included in their system, their fields, publications collected, etc. Regarding their indicators, often they are not technically described (e.g. the RG score), and their potential biases, limitations and technical problems are unknown to their users. This is in conflict with common practices in scientometrics, and as stated in the *Leiden Manifesto* (Hicks et al. 2015) one should “[k]eep data collection and analytical processes open, transparent and simple”, particularly when evaluating individual scholars.

### Lack of validity and reliability of the data and indicators provided

None of these sources has been validated in their individual-level data. Information about how they deal with the traditional issues of homonymy and synonymy is missing. This limitation also applies to sources that are user-maintained (e.g. Google Scholar or Research Gate), as often they automatically update their user profiles or they can be updated by users different than the intended scholar, in any case biasing the use of their data and indicators towards scholars with more up-to-date profiles. Indicators like the h-index are usually uncritically incorporated in these systems, ignoring issues related with their accuracy and usefulness. These sources also fail in incorporating any individual context (e.g. age, gender, mobility, education, country, etc.), and generally do not account for field differences in scholarly practices. Manipulation or gaming is possible and easy (López-Cózar et al. 2014) but usually ignored.

## On the cautionary use of the (multiple versions of the) h-index

In this section we develop some specific warnings regarding the existence of different online versions of the h-index for their use in research evaluation. We will frame these warnings however around the fundamental challenges around the h-index (and its massive dissemination across multiple online platforms) depicted in previous sections. It is of course not possible in this short letter to present all the important challenges, but we will try here to introduce at least some of them.

The most important challenge of the h-index is that, like essentially any other single indicator, it introduces a particular notion of scientific performance as ideal (as shown above). Researchers with larger outputs of not necessarily higher impact are preferred over more selective ones. The h-index is a size-dependent indicator, therefore it is intertwined with the output size, age, career length or collaboration networks of scholars, leading to higher scores for more senior and prolific scholars. As a size-dependent indicator it is directly related to indicators such as P and C, capturing a similar dimension of scientific performance, but with the disadvantage that the h-index violates certain basic consistency criteria (Waltman and Van Eck 2012).

The bibliometric analysis of individual scholars is one of the most contested and challenging issues in scientometrics (Benedictus et al. 2016; Costas e al. 2010; Wildgaard et al. 2014). At the individual level, indicators show a lower validity, and data collection and coverage issues are more critical (as partly shown by TSD). Moreover, the complexity and diversity of aspects that need to be taken into account when evaluating individual scholars is not met by the use of any single bibliometric indicator. Jorge Hirsch in his seminal paper on the h-index argued that “[o]bviously, a single number can never give more than a rough approximation to an individual’s multifaceted profile, and many other factors should be considered in combination in evaluating an individual”. The *Leiden Manifesto* also highlights the importance of not relying solely on bibliometrics and single indicators. It seems that these warnings have been often overlooked in favor of the (perceived) simplistic value of the h-index, and get exacerbated with the profusion of indicators across multiple online platforms, by for example overlooking the black box nature of sources like Google Scholar or ResearchGate (Wouters and Costas 2012).

The proliferation of online platforms that take the individual scholar as the primary evaluative object creates the perception that individual researchers are indisputable objects of measurement, systematically turning them into quantified ‘academic selves’ (Hammarfelt et al. 2016). However, this massive availability of multiple online sources of individual scholars data should not be seen as an endorsement for the use and application of these data and indicators at the individual level, and particularly does not mean that the individual level is a more suitable level of evaluation of scientific performance than other levels such as the group, department, faculty, university or even country levels. Besides, in addition to the coverage, data quality and transparency issues of these platforms, the uncritical incorporation of the h-index from these online platforms in formal academic evaluations may create even more problematic situations in which the biases and limitations of these sources and indicators are also incorporated into the evaluations, potentially creating new additional problems in research evaluation (e.g. unfairness, manipulability, etc.).

## Final considerations

TSD’s letter reminds us of the multiple problems and issues related to the h-index, with a special focus on the distorting effect caused by the proliferation of sources that provide this indicator. However we miss in TSD’s letter a stronger criticism and a more thorough discussion of the more fundamental problems related with this profusion of sources and h-indexes at the individual level. We tried here to provide a stronger criticism, pointing to some of these fundamental conceptual and methodological issues.

Bibliometric indicators applied to individuals are powerful tools for studying scholars’ interactions, their demographics, gender, careers, mobility, etc. (Costas et al. 2010). Science is grounded in a global collaborative effort, in which a vast *eco*-*system* of scholars interact and produce new scientific knowledge. Using indicators to understand this eco-system and developing more multidimensional and contextualized evaluations of scientific performance are more useful and reasonable approaches. Here is where we believe indicators at the level of individual scholars have most value, much more than in just ranking individuals by their Google Scholar h-index or any other one-dimensional bibliometric statistic.
